# Supplementary material for: Cryo-EM Structures Reveal Upstream DNA Interactions within the Mitochondrial Transcription Initiation Complex
Source: bioRxiv. 2026 Jun 24:2026.04.09.717523. Originally published 2026 Apr 11. Preprint. [Version 2] doi: 10.64898/2026.04.09.717523 (PMC13089591; doi:10.64898/2026.04.09.717523)
Supplement: Supplement 1 [file media-1.pdf]

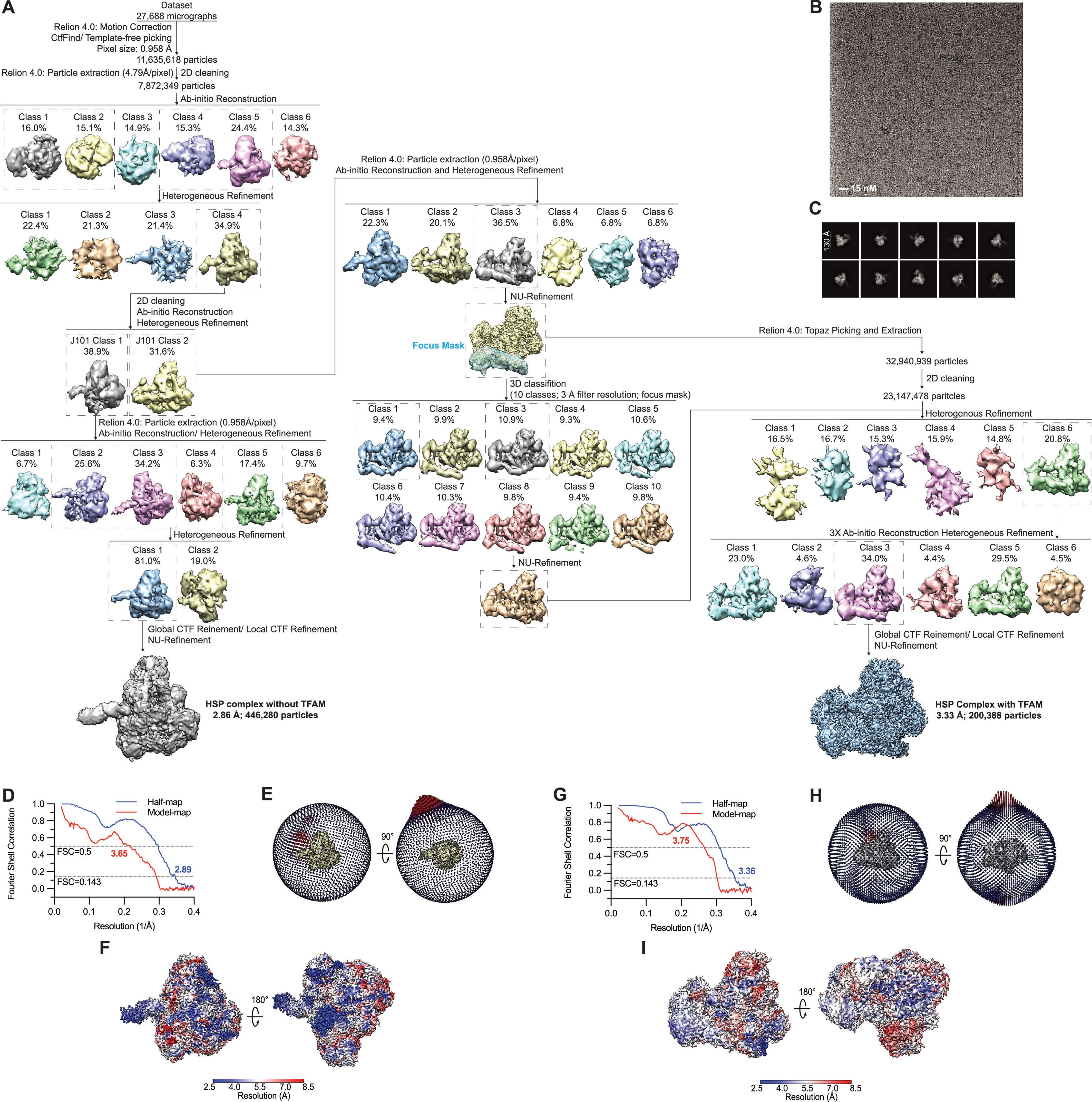
A
Dataset
27,688 micrographs
Relion 4.0: Motion Correction
CtfFind/ Template-free picking
Pixel size: 0.958 Å
11,635,618 particles
Relion 4.0: Particle extraction (4.79Å/pixel)
2D cleaning
7,872,349 particles
Ab-initio Reconstruction
Class 1 16.0%
Class 2 15.1%
Class 3 14.9%
Class 4 15.3%
Class 5 24.4%
Class 6 14.3%
Heterogeneous Refinement
Class 1 22.4%
Class 2 21.3%
Class 3 21.4%
Class 4 34.9%
2D cleaning
Ab-initio Reconstruction
Heterogeneous Refinement
J101 Class 1 38.9%
J101 Class 2 31.6%
Relion 4.0: Particle extraction (0.958Å/pixel)
Ab-initio Reconstruction/ Heterogeneous Refinement
Class 1 6.7%
Class 2 25.6%
Class 3 34.2%
Class 4 6.3%
Class 5 17.4%
Class 6 9.7%
Heterogeneous Refinement
Class 1 81.0%
Class 2 19.0%
Global CTF Reinement/ Local CTF Refinement
NU-Refinement
HSP complex without TFAM
2.86 Å; 446,280 particles
Relion 4.0: Particle extraction (0.958Å/pixel)
Ab-initio Reconstruction and Heterogeneous Refinement
Class 1 22.3%
Class 2 20.1%
Class 3 36.5%
Class 4 6.8%
Class 5 6.8%
Class 6 6.8%
NU-Refinement
Focus Mask
3D classifition
(10 classes; 3 Å filter resolution; focus mask)
Class 1 9.4%
Class 2 9.9%
Class 3 10.9%
Class 4 9.3%
Class 5 10.6%
Class 6 10.4%
Class 7 10.3%
Class 8 9.8%
Class 9 9.4%
Class 10 9.8%
NU-Refinement
Relion 4.0: Topaz Picking and Extraction
32,940,939 particles
2D cleaning
23,147,478 paritcles
Heterogenous Refinement
Class 1 16.5%
Class 2 16.7%
Class 3 15.3%
Class 4 15.9%
Class 5 14.8%
Class 6 20.8%
3X Ab-initio Reconstruction Heterogeneous Refinement
Class 1 23.0%
Class 2 4.6%
Class 3 34.0%
Class 4 4.4%
Class 5 29.5%
Class 6 4.5%
Global CTF Reinement/ Local CTF Refinement
NU-Refinement
HSP Complex with TFAM
3.33 Å; 200,388 particles
B
15 nM
C
130 Å
D
Half-map
Model-map
Fourier Shell Correlation
1.0
0.8
0.6
0.4
0.2
0.0
FSC=0.5
FSC=0.143
3.65
2.89
0.0
0.1
0.2
0.3
0.4
Resolution (1/Å)
E
90°
G
Half-map
Model-map
Fourier Shell Correlation
FSC=0.5
FSC=0.143
3.75
3.36
Resolution (1/Å)
H
90°
F
180°
2.5
4.0
5.5
7.0
8.5
Resolution (Å)
I
180°
2.5
4.0
5.5
7.0
8.5
Resolution (Å)

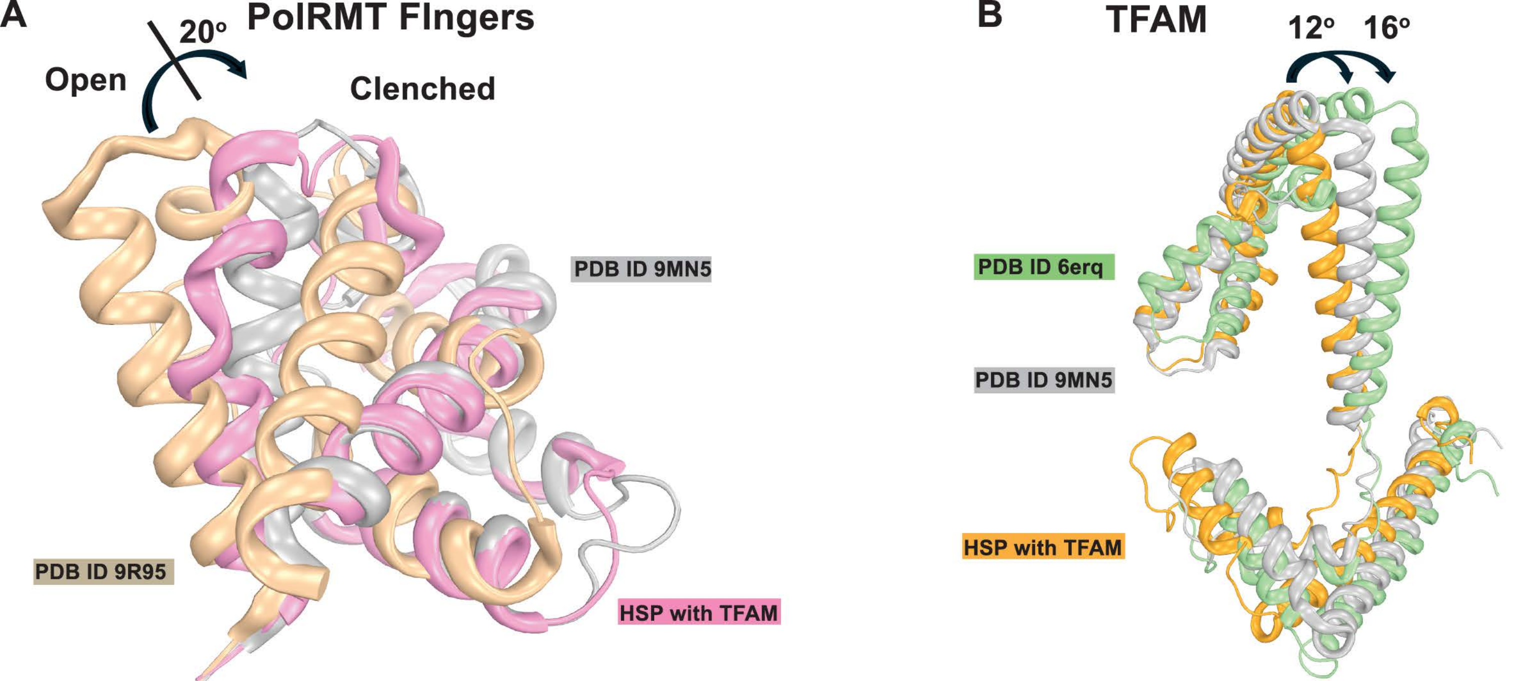
A
PolRMT FIngers
20°
Open
Clenched
PDB ID 9MN5
PDB ID 9R95
HSP with TFAM
B
TFAM
12°
16°
PDB ID 6erq
PDB ID 9MN5
HSP with TFAM

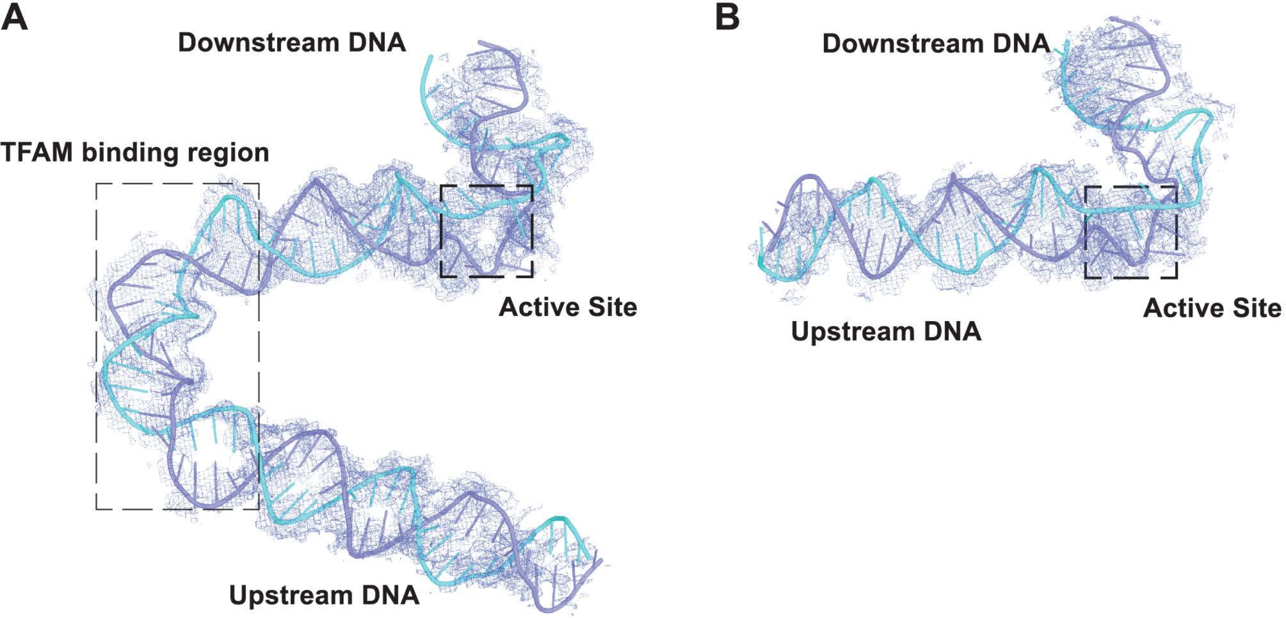

A
Downstream DNA
TFAM binding region
Active Site
Upstream DNA
B
Downstream DNA
Active Site
Upstream DNA

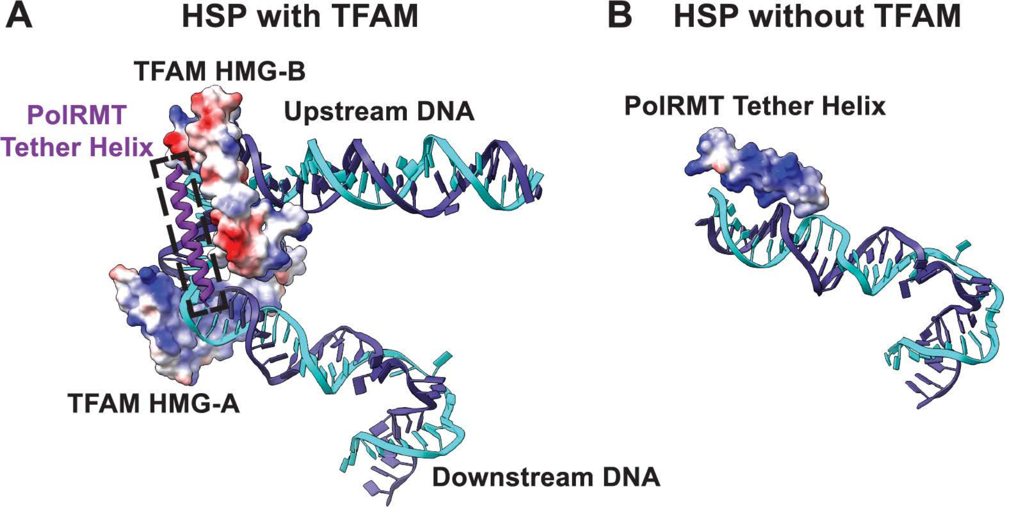
A
HSP with TFAM
B
HSP without TFAM
TFAM HMG-B
PolRMT
Tether Helix
Upstream DNA
TFAM HMG-A
Downstream DNA
PolRMT Tether Helix

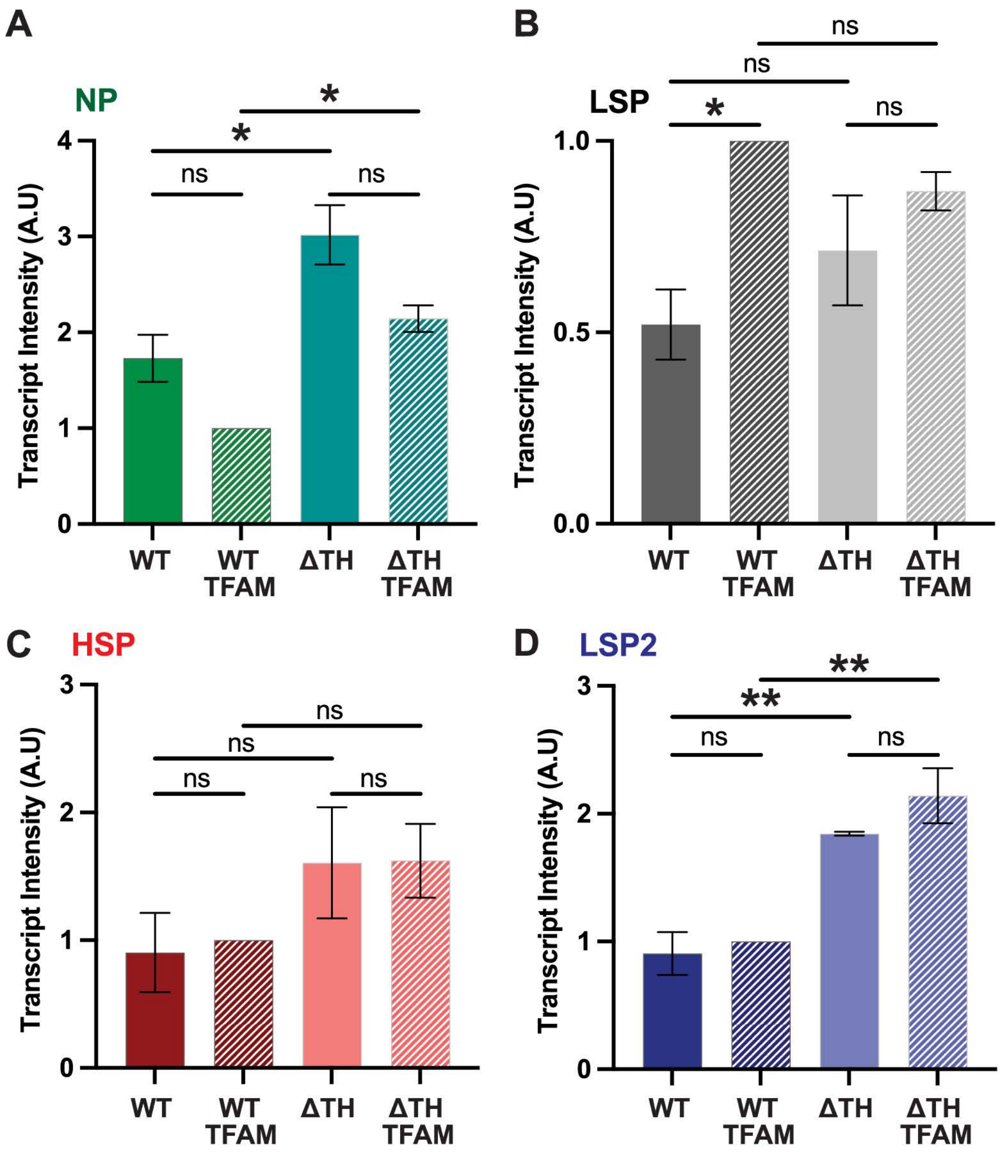
A
NP
Transcript Intensity (A.U)
0
1
2
3
4
ns
*
*
ns
WT
WT TFAM
ΔTH
ΔTH TFAM
B
LSP
Transcript Intensity (A.U)
0.0
0.5
1.0
*
ns
ns
ns
WT
WT TFAM
ΔTH
ΔTH TFAM
C
HSP
Transcript Intensity (A.U)
0
1
2
3
ns
ns
ns
ns
WT
WT TFAM
ΔTH
ΔTH TFAM
D
LSP2
Transcript Intensity (A.U)
0
1
2
3
ns
**
**
ns
WT
WT TFAM
ΔTH
ΔTH TFAM

A

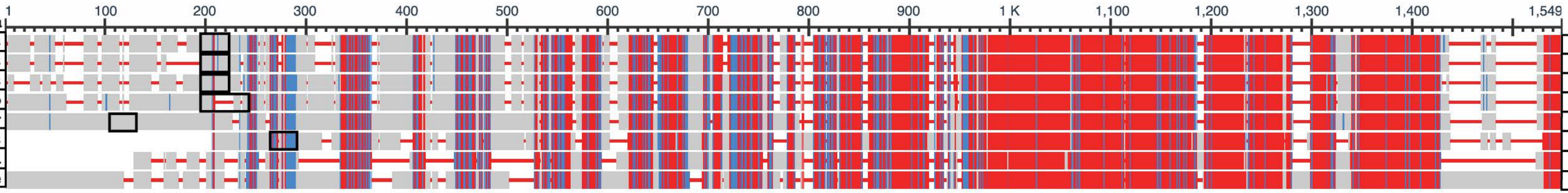

Organism	AA	UniProt ID
Homo Sapiens	1,230	O00411
Mus Musculus	1,207	Q3U3J3
Gallus Gallus	1,236	E1BZ13
Danio Rerio	1,281	A0ACD6B7G4
Drosphila Melanogaster	1,369	Q9VPW4
Caenorhabditis Elegans	1,138	Q8WQA3
Arabidopsis Thaliana	1,011	Q9LFV6
Saccharomyces Cerevisiae	1,351	P13433

B

Organism	Potential Tether Helix Sequence	Position
Homo Sapiens	WAKILEKDKRTQQMRMQRLKAKLQM	122-146
Mus Musculus	WAQKLEAEKRVKQRRQKEVDQQKQA	97-121
Gallus Gallus	WTEKLKKEMYIRQLKVEKKLSIAAS	108-132
Danio Rerio	WMAKLNREMWINPKTKTSKKVIKTG	156-180
Drosphila Melanogaster	VELLEVTEQAISERRSRVKRLKSKK	104-128*
Caenorhabditis Elegans	KQKKRGIWRQRVKQIALADIVRFTL	59-83*
Arabidopsis Thaliana	N/A	
Saccharomyces Cerevisiae	N/A	

C

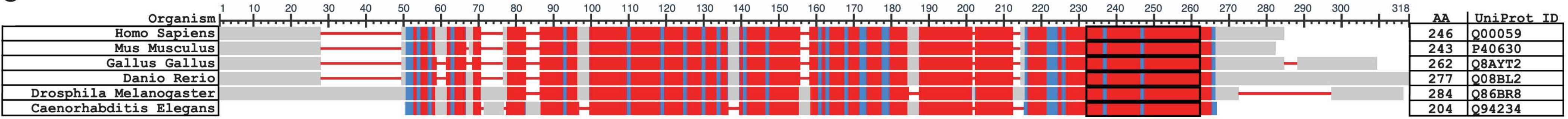

Organism	AA	UniProt ID
Homo Sapiens	246	Q00059
Mus Musculus	243	P40630
Gallus Gallus	262	Q8AYT2
Danio Rerio	277	Q08BL2
Drosphila Melanogaster	284	Q86BR8
Caenorhabditis Elegans	204	Q94234

D

Organism	TFAM HMG-B Helix 3
Homo Sapiens	DSEKELYIQHAKEDETRYHNEMKSWEEQMIE
Mus Musculus	PEEKQAYIQLAKDDRIRYDNEMKSWEEQMAE
Gallus Gallus	SSQKQPYLQLAQDDKVRYQNEMKSWEAKMVE
Danio Rerio	DTQKQMYIQLAEDDKVRYKNEIKSWEEHMME
Drosphila Melanogaster	DSEKEVYMQESRKEMELYRKAISVWEEKMIR
Caenorhabditis Elegans	DSQKKKYTDEAKKLKDEYHVVLQKWEAEQKE
