# Supplementary material for: Structural Basis of Mitochondrial Transcription Regulation via Interactions of PolRMT and TFAM with Upstream Promoter DNA
Source: bioRxiv. 2026 Apr 11:2026.04.09.717523. Preprint. [Version 1] doi: 10.64898/2026.04.09.717523 (PMC13089591; doi:10.64898/2026.04.09.717523)
Supplement: Supplementary file 1 [file NIHPP2026.04.09.717523V1-supplement-1.pdf]

bioRxiv preprint doi: https://doi.org/10.64898/2026.04.09.717523; this version posted April 12, 2026. The copyright holder for this preprint (which was not certified by peer review) is the author/funder, who has granted bioRxiv a license to display the preprint in perpetuity. It is made available under aCC-BY 4.0 International license.

Supplementary Data

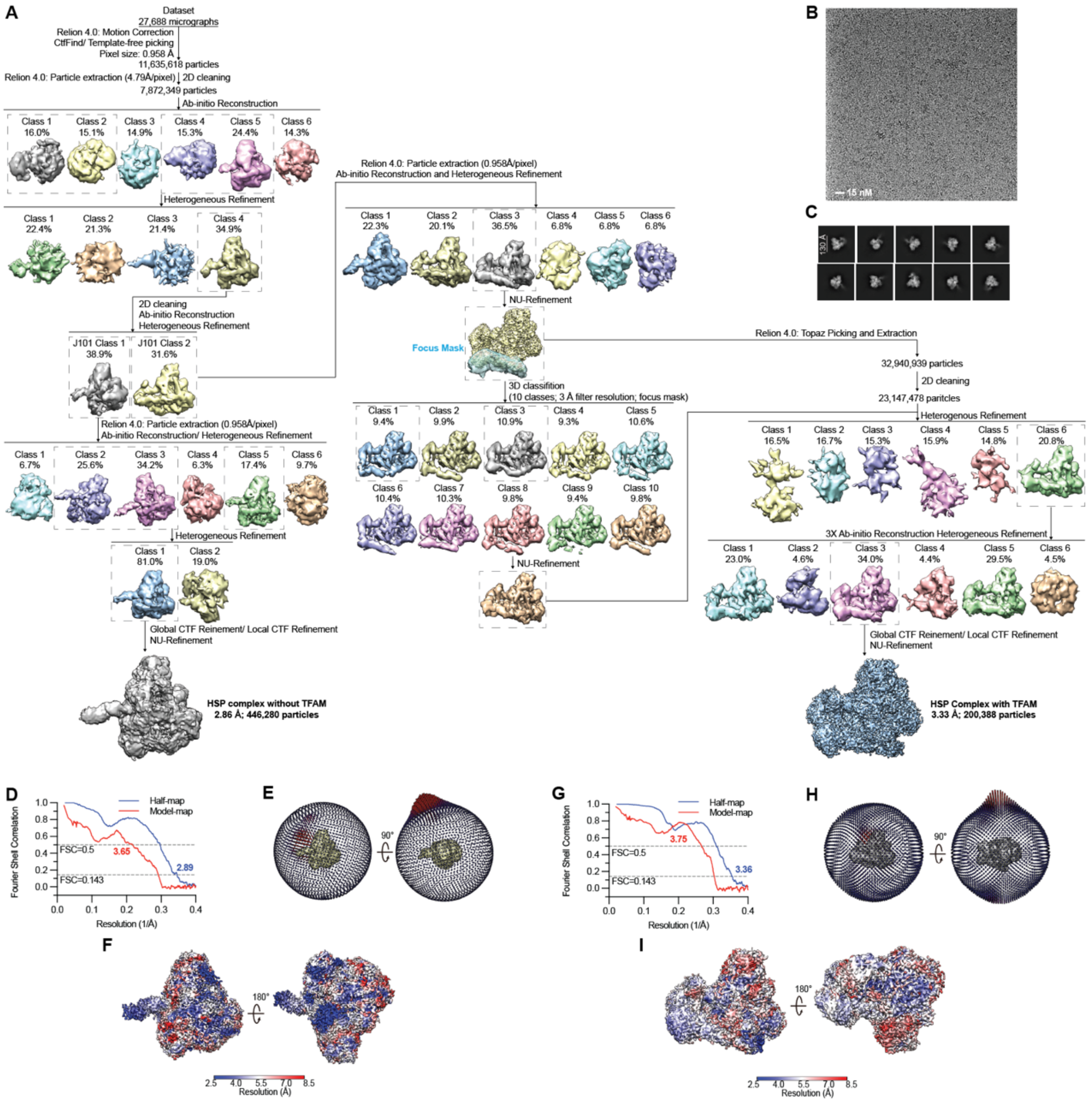

Figure S1. Cryo-EM reconstruction of the mtTIC on HSP with and without TFAM, related to **Figure 1** and **4.** (A) Overview of image processing and refinement strategy. Steps performed in RELION are indicated, and all other steps were performed with CryoSPARC-4.2. (B) Representative cryo-EM micrograph. (C) Representative 2D class averages. (D) Gold-standard Fourier shell correlation (FSC) curve of the HSP without TFAM complex between the two half maps, with indicated resolution at FSC=0.143 shown in blue. FSC curve between the refined model and the cryo-EM map, with indicated resolution at FSC=0.5 shown in red. (E) The angular distribution of the particles used in the final 3D reconstruction of the HSP without TFAM complex. (F) Local resolution of the cryo-EM density map of the HSP without TFAM complex. (G) Gold-standard FSC curve of the HSP with TFAM complex between the two half maps, with indicated resolution at FSC=0.143 shown in blue. FSC curve between the refined model and the cryo-EM map, with indicated

bioRxiv preprint doi: https://doi.org/10.64898/2026.04.09.717523; this version posted April 12, 2026. The copyright holder for this preprint (which was not certified by peer review) is the author/funder, who has granted bioRxiv a license to display the preprint in perpetuity. It is made available under aCC-BY 4.0 International license.

resolution at FSC=0.5 shown in red. (H) The angular distribution of the particles used in the final 3D reconstruction of the HSP with TFAM complex. (I) Local resolution of the cryo-EM density map of the HSP with TFAM complex.

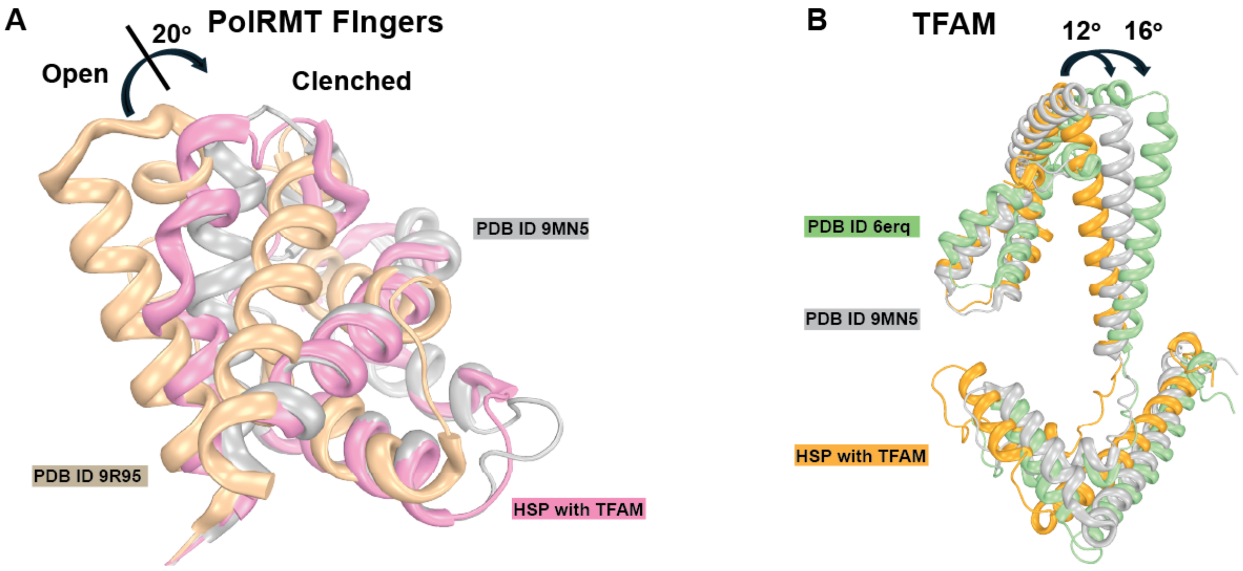

Figure S2. Comparison of our HSP with TFAM structure to other mtTIC structures. (A) Conformation of PolRMT fingers domain from our HSP with TFAM structure in pink compared with the LSP TIC0 structure in gray (PDB: 9MN5) (clenched) and the LSP pre-IC3 with incoming GTP in wheat (PDB: 9R95) (open). From the open to clenched conformation, the fingers domain rotates 20º toward the active site. Structures were aligned by the Cα atoms of the conserved PolRMT palm domain. (B) Rotation of TFAM in the mtTICs. Relative to the LSP TIC0 structure in gray (PDB: 9MN5) our HSP structure (orange) is rotated 12º, and 16º relative to the HSP crystal structure in pale green (PDB: 6ERQ). Structures were aligned by the Cα atoms of the conserved PolRMT and TFB2M core.

bioRxiv preprint doi: https://doi.org/10.64898/2026.04.09.717523; this version posted April 12, 2026. The copyright holder for this preprint (which was not certified by peer review) is the author/funder, who has granted bioRxiv a license to display the preprint in perpetuity. It is made available under aCC-BY 4.0 International license.

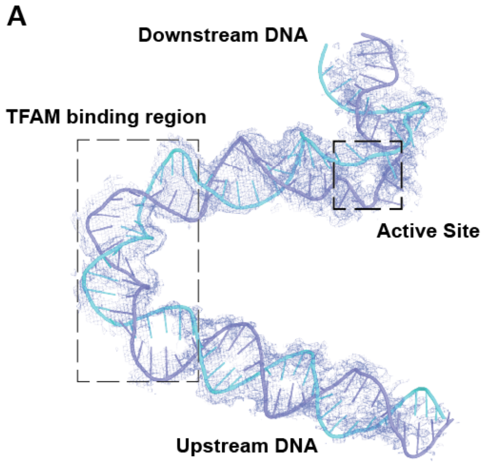

Figure S3. Trajectory of upstream DNA in the HSP with and without TFAM complexes. (A) In the HSP with TFAM complex, TFAM bends the upstream DNA proximal to the active site into a U-turn shape and the more distal upstream DNA extends linearly after the bend. (B) In the HSP without TFAM complex, only a short portion of the upstream DNA is observed, which is linear in the absence of TFAM-induced bending.

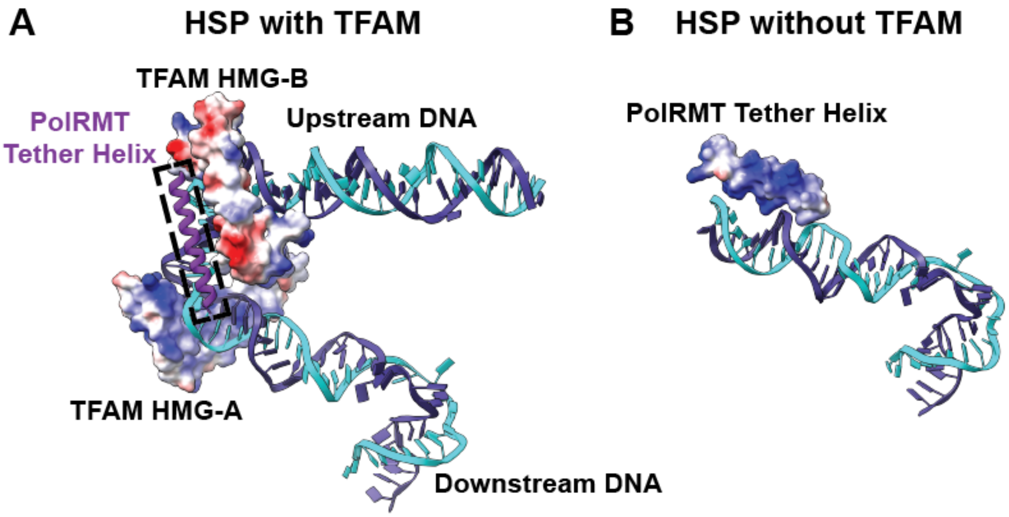

Figure S4. Electrostatic interactions of the PolRMT tether helix in the HSP with or without TFAM complexes. (A) Electrostatic surface of TFAM and cartoon representation of the PolRMT tether helix in the HSP with TFAM complex. The highly positively charged surface of the tether helix engages the negatively charged helix 3 of the HMG-B box. (B) Electrostatic surface of the positively charged PolRMT tether helix near the negatively charged DNA backbone in the HSP without TFAM complex.

bioRxiv preprint doi: https://doi.org/10.64898/2026.04.09.717523; this version posted April 12, 2026. The copyright holder for this preprint (which was not certified by peer review) is the author/funder, who has granted bioRxiv a license to display the preprint in perpetuity. It is made available under aCC-BY 4.0 International license.

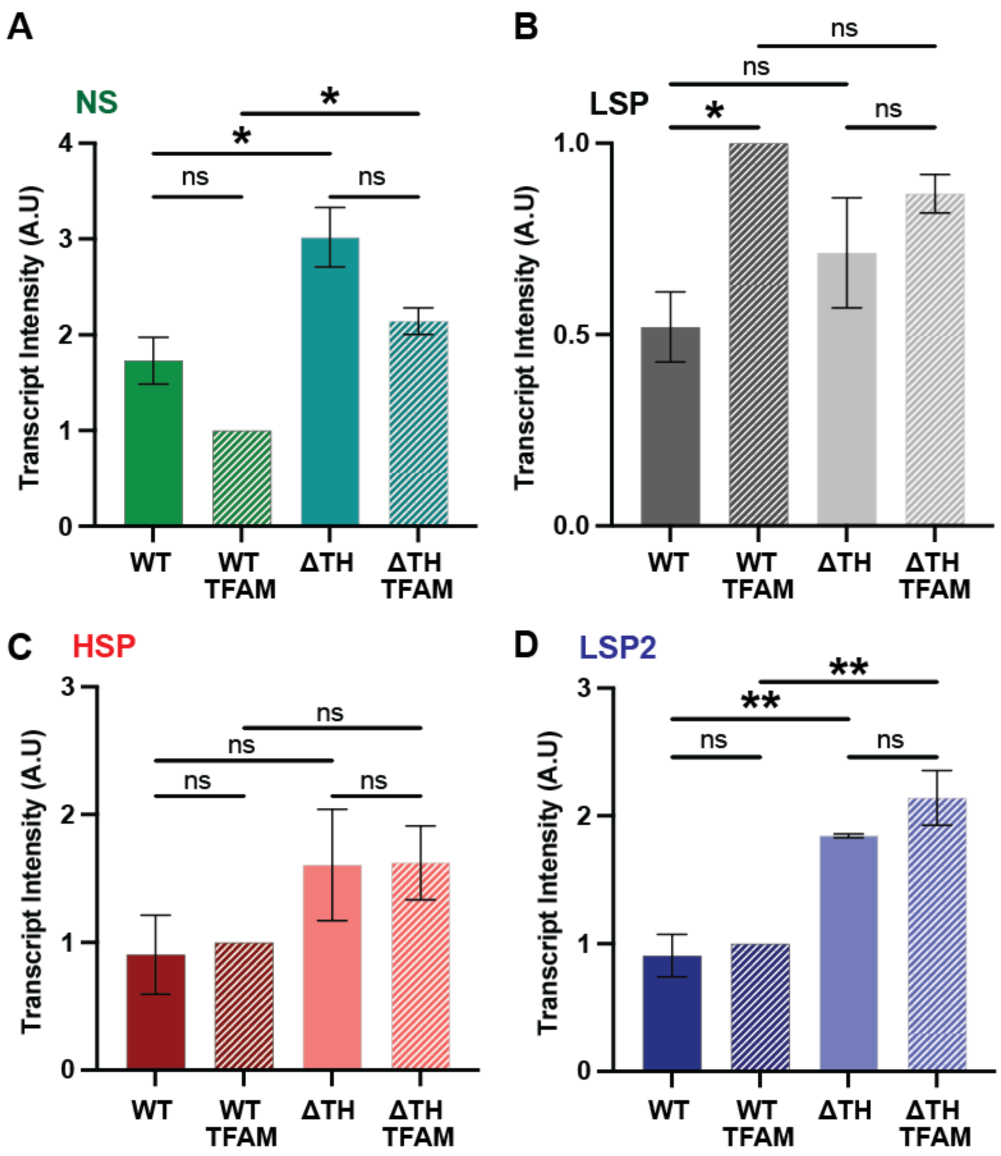

Figure S5. The auto-inhibitory effect of the PolRMT tether helix follows a similar but less pronounced trend at higher PolRMT concentration, related to **Figure 4**. Transcription reactions contained 20 nM DNA template, 100 nM TFB2M, 0 or 60 nM TFAM, and 0 or 300 nM PolRMT. (A) Quantification of transcript production from the NS DNA substrate. (B) Quantification of transcript production from the LSP. (C) Quantification of transcript production from the HSP. (D) Quantification of transcript production from the LSP2. Mean and SEM were determined from 3 independent replicates. Significance was determined using a 1-way ANOVA test with Tukey's multiple comparisons test. Significance: * p<0.05, ** p<0.01, **** p<0.0001.

bioRxiv preprint doi: https://doi.org/10.64898/2026.04.09.717523; this version posted April 12, 2026. The copyright holder for this preprint (which was not certified by peer review) is the author/funder, who has granted bioRxiv a license to display the preprint in perpetuity. It is made available under aCC-BY 4.0 International license.

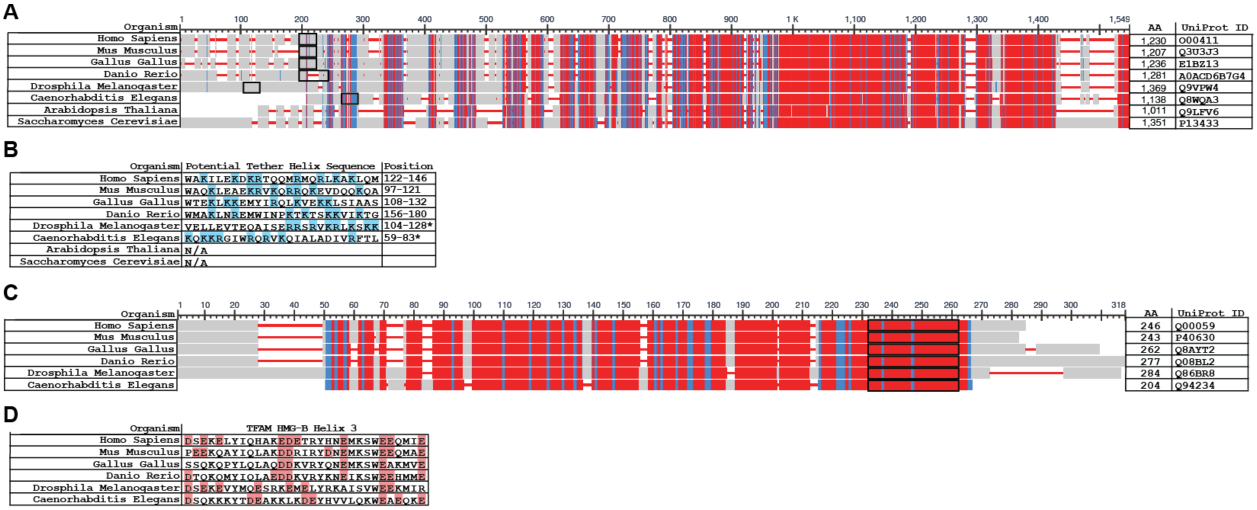

Figure S6. Sequence and charge conservation of the PolRMT tether helix and TFAM HMG-B box helix 3. (A) Alignment of PolRMT and its homologs from various eukaryotic species. Red shading indicates high sequence conservation, blue shading indicates lower sequence conservation, and black outline boxes indicate location of probable tether helix sequences. (B) Sequences of the probable tether helix sequences with blue boxes indicating the positively charged residues, * indicates sequences that did not align well with the human PolRMT tether helix. (C) Alignment of TFAM and its homologs from metazoan species that utilize TFAM as part of a three-component mtTIC. Red shading indicates high sequence conservation, blue shading indicates lower sequence conservation, and black outline boxes indicate the HMG-B box helix 3. (D) Sequences of the TFAM HMG-B box helix 3 with red boxes indicating the negatively charged residues.

bioRxiv preprint doi: https://doi.org/10.64898/2026.04.09.717523; this version posted April 12, 2026. The copyright holder for this preprint (which was not certified by peer review) is the author/funder, who has granted bioRxiv a license to display the preprint in perpetuity. It is made available under aCC-BY 4.0 International license.

Table S1. Oligonucleotides for PolRMT cloning and *in vitro* transcription templates.

Oligo Name	Description	Sequence (5' to 3')
HSP 60 NT	HSP NT -60 to +11, mutated from -4 to +3 to create bubble	CCATCCTACCCAGCACACACACACCGCTGCTAACC CCATACCCCGAACCAACCAAATTATCCCGACACCCC
HSP 60 TS	HSP TS -60 to +11	GGGGTGTCTTTGGGGTTTGGTTGGTTCGGGGTATG GGGTTAGCAGCGGTGTGTGTGTGCTGGGTAGGATG G
LSP 40 NT	LSP NT -40 to +20	GTGTTAGTTGGGGGGTGACTGTTAAAAGTGCATACC GCCAAAAGATAAAATTTGAAATCT
LSP 40 TS	LSP TS -40 to +20	AGATTTCAAATTTTATCTTTTGGCGGTATGCACTTTTA ACAGTCACCCCCCAACTAACAC
LSP 70 NT	LSP NT -70 to +20	AGTAGTATGGGAGTGGGAGGGGAAAATAATGTGTTA GTTGGGGGGTGACTGTTAAAAGTGCATACCGCCAA AAGATAAAATTTGAAATCT
LSP 70 TS	LSP TS -70 to +20	AGATTTCAAATTTTATCTTTTGGCGGTATGCACTTTTA ACAGTCACCCCCCAACTAACACATTATTTTCCCCTC CCACTCCCATACTACT
HSP 40 NT	HSP NT -40 to +20	ACACCGCTGCTAACCCCATACCCCGAACCAACCAAA CCCCAAAGACACCCCCCACAGTTT
HSP 40 TS	HSP TS -40 to +20	AAACTGTGGGGGGTGTCTTTGGGGTTTGGTTGGTT CGGGGTATGGGGTTAGCAGCGGTGT
HSP 70 NT	HSP NT -70 to +20	CAACCCCCGCCCATCCTACCCAGCACACACACACC GCTGCTAACCCCATACCCCGAACCAACCAAACCCC AAAGACACCCCCCACAGTTT
HSP 70 TS	HSP TS -70 to +20	AAACTGTGGGGGGTGTCTTTGGGGTTTGGTTGGTT CGGGGTATGGGGTTAGCAGCGGTGTGTGTGTGCTG GGTAGGATGGGCGGGGGTTG
LSP2 40 NT	LSP2 NT -40 to +20	GAGTCAATACTTGGGTGGTACCCAAATCTGCTTCCC CATGAAAGAACAGAGAATAGTTTA
LSP2 40 TS	LSP2 TS -40 to +20	TAAACTATTCTCTGTTCTTTCATGGGGAAGCAGATTT GGGTACCACCCAAGTATTGACTC
LSP2 70 NT	LSP2 NT -70 to +20	GTACGAAATACATAGCGGTTGTTGATGGGTGAGTCA ATACTTGGGTGGTACCCAAATCTGCTTCCCCATGAA AGAACAGAGAATAGTTTA
LSP2 70 TS	LSP2 TS -70 to +20	TAAACTATTCTCTGTTCTTTCATGGGGAAGCAGATTT GGGTACCACCCAAGTATTGACTCACCCATCAACAAC CGCTATGTATTTCGTAC
NS 70 NT	NS NT -70 to +20	TATATGATATGTCTCCATACCCATTACAATCTCCAGCA TTCCCCCTCAAACCTAAGAAATATGTCTGATAAAAGA GTTACTTTGATAGAG

bioRxiv preprint doi: https://doi.org/10.64898/2026.04.09.717523; this version posted April 12, 2026. The copyright holder for this preprint (which was not certified by peer review) is the author/funder, who has granted bioRxiv a license to display the preprint in perpetuity. It is made available under aCC-BY 4.0 International license.

	mtDNA sequence 4211-4300	
NS 70 TS	NS TS -70 to +20 mtDNA sequence 4211-4300	CTCTATCAAAGTAACTCTTTTATCAGACATATTTCTTAGGTTTGAGGGGGAATGCTGGAGATTGTAATGGGTATGGAGACATATCATATA
ES 42 NT	NT -42 to +10 with -9 to -1 bubble for elongation	GCACTCACAGTCGCATCATAATCGCTCAACGATTAATTATACACGTAGCC
ES 42 TS	TS -42 to +10 for elongation	GGCTACGTGTCGCCGGGCGCGTTGAGCGATTATGATGCGACTGTGAGTGC
ES 72 NT	NT -72 to +10 with -9 to -1 bubble for elongation	ACTATTCTGCCTAGCAAACTCAAACTACGAACGCACTCACAGTCGCATCATAATCGCTCAACGATTAATTATACACGTAGCC
ES 72 TS	TS -72 to +10 for elongation	GGCTACGTGTCGCCGGGCGCGTTGAGCGATTATGATGCGACTGTGAGTGCGTTCGTAGTTTGAGTTTGCTAGGCAGAATAGT
ES RNA	FAM-labeled RNA pairs with the TS from -9 to -1 with a 3-nt overhang on the 5' end	/56-FAM/ rUrUrUrCrGrCrCrCrGrGrCrG
PolRMT 3KE Fwd	PolRMT Mutation K425E, K428E, K432E	AAGTGGAGCATGCACGCGAGACCCTGAAAACCTTACGTGATCAGT
PolRMT 3KE Rev	PolRMT Mutation K425E, K428E, K432E	GTGCATGCTCCACTTCCTCACTCGGCAGGGTCGGTTT
PolRMT ΔTH Fwd	PolRMT truncation of residues 122-146	CCGTTTCAGAGTGGCG
PolRMT ΔTH Rev	PolRMT truncation of residues 122-146	ACGACCACACGGCAC

bioRxiv preprint doi: https://doi.org/10.64898/2026.04.09.717523; this version posted April 12, 2026. The copyright holder for this preprint (which was not certified by peer review) is the author/funder, who has granted bioRxiv a license to display the preprint in perpetuity. It is made available under aCC-BY 4.0 International license.

Table S2. Structure alignment of the HSP with TFAM TIC core to other mtTIC structures. Structures were aligned by the Cα atoms of the conserved PolRMT and TFB2M core.

PDB ID	Description	Aligned Atoms (#)	RMSD (Å)	Rotation of TFAM (º)
6ERQ	HSP TIC0 crystal structure	1106	1.135	15.9
6ERP	LSP TIC0 crystal structure	1110	1.108	16.7
9MN5	LSP IC0	893	0.857	11.8
9MN4	LSP IC3 with 3-nt RNA	850	0.895	14.1
9GZM	LSP pre-IC3 with 2-nt RNA and incoming GTP	1159	0.856	7.2
9R95	LSP slipped IC3 with 2-nt RNA, ATP, and incoming GTP	1136	0.716	6.4
9R96	LSP slipped pre-IC4 with 2-nt RNA, dATP, and incoming GTP	1149	0.837	5.7
9GZN	LSP pre-IC3 with 2-nt RNA, incoming GTP, and no TFAM	1140	0.901	N/A
9GZO	LSP slipped IC3 with 2-nt RNA, ATP, incoming GTP, and no TFAM	1136	1.023	N/A
11PS	HSP without TFAM	1331	0.601	N/A

bioRxiv preprint doi: https://doi.org/10.64898/2026.04.09.717523; this version posted April 12, 2026. The copyright holder for this preprint (which was not certified by peer review) is the author/funder, who has granted bioRxiv a license to display the preprint in perpetuity. It is made available under aCC-BY 4.0 International license.

Table S3. Structure alignment of the HSP without TFAM TIC core to other mtTIC structures. Structures were aligned by the Cα atoms of the conserved PolRMT and TFB2M core.

PDB ID	Description	Aligned Atoms (#)	RMSD (Å)
6ERQ	HSP TIC0 crystal structure	1117	1.290
6ERP	LSP TIC0 crystal structure	1118	1.259
9MN5	LSP IC0	889	0.912
9MN4	LSP IC3 with 3-nt RNA	836	1.011
9GZM	LSP pre-IC3 with 2-nt RNA and incoming GTP	1185	1.187
9R95	LSP slipped IC3 with 2-nt RNA, ATP, and incoming GTP	1181	1.074
9R96	LSP slipped pre-IC4 with 2-nt RNA, dATP, and incoming GTP	1181	1.148
9GZN	LSP pre-IC3 with 2-nt RNA, incoming GTP, and no TFAM	1180	1.201
9GZO	LSP slipped IC3 with 2-nt RNA, ATP, incoming GTP, and no TFAM	1159	1.244
11PR	HSP with TFAM	1331	0.601
